# Accuracy of commercial geocoding: assessment and implications

**DOI:** 10.1186/1742-5573-3-8

**Published:** 2006-07-20

**Authors:** Eric A Whitsel, P Miguel Quibrera, Richard L Smith, Diane J Catellier, Duanping Liao, Amanda C Henley, Gerardo Heiss

**Affiliations:** 1 Departments of Epidemiology and Medicine, University of North Carolina, Cardiovascular Disease Program, Bank of America Center Suite 306, 137 East Franklin Street, Chapel Hill, NC 27514, USA; 2 Department of Epidemiology, University of North Carolina, Cardiovascular Disease Program, Bank of America Center Suite 306, 137 East Franklin Street, Chapel Hill, NC 27514, USA; 3 Department of Statistics and Operations Research, University of North Carolina, 201 Smith Building 128, Chapel Hill, NC 27599, USA; 4 Department of Biostatistics, University of North Carolina, Collaborative Studies Coordinating Center, 137 East Franklin Street, Chapel Hill, NC 27514, USA; 5 Department of Health Evaluation Sciences, Pennsylvania State University College of Medicine, 600 Centerview Drive Suite 2200, A210, Hershey, PA 17033, USA; 6 Walter Royal Davis Library, University of North Carolina, Reference Department, Geographic Information Services, Chapel Hill, NC 27599, USA

## Abstract

**Background:**

Published studies of geocoding accuracy often focus on a single geographic area, address source or vendor, do not adjust accuracy measures for address characteristics, and do not examine effects of inaccuracy on exposure measures. We addressed these issues in a Women's Health Initiative ancillary study, the Environmental Epidemiology of Arrhythmogenesis in WHI.

**Results:**

Addresses in 49 U.S. states (n = 3,615) with established coordinates were geocoded by four vendors (A-D). There were important differences among vendors in address match rate (98%; 82%; 81%; 30%), concordance between established and vendor-assigned census tracts (85%; 88%; 87%; 98%) and distance between established and vendor-assigned coordinates (mean ***ρ ***[meters]: 1809; 748; 704; 228). Mean ***ρ ***was lowest among street-matched, complete, zip-coded, unedited and urban addresses, and addresses with North American Datum of 1983 or World Geodetic System of 1984 coordinates. In mixed models restricted to vendors with minimally acceptable match rates (A-C) and adjusted for address characteristics, within-address correlation, and among-vendor heteroscedasticity of ***ρ***, differences in mean ***ρ ***were small for street-type matches (280; 268; 275), i.e. likely to bias results relying on them about equally for most applications. In contrast, differences between centroid-type matches were substantial in some vendor contrasts, but not others (5497; 4303; 4210) p_interaction _< 10^-4^, i.e. more likely to bias results differently in many applications. The adjusted odds of an address match was higher for vendor A versus C (odds ratio = 66, 95% confidence interval: 47, 93), but not B versus C (OR = 1.1, 95% CI: 0.9, 1.3). That of census tract concordance was no higher for vendor A versus C (OR = 1.0, 95% CI: 0.9, 1.2) or B versus C (OR = 1.1, 95% CI: 0.9, 1.3). Misclassification of a related exposure measure – distance to the nearest highway – increased with mean ***ρ ***and in the absence of confounding, non-differential misclassification of this distance biased its hypothetical association with coronary heart disease mortality toward the null.

**Conclusion:**

Geocoding error depends on measures used to evaluate it, address characteristics and vendor. Vendor selection presents a trade-off between potential for missing data and error in estimating spatially defined attributes. Informed selection is needed to control the trade-off and adjust analyses for its effects.

## Background

Various sources of measurement error have substantial implications for the accuracy of epidemiologic estimates. Exposure measurement error, for example, may arise when geographic information systems are trusted without recognizing the limitations of processes that rely on them. One such process is address matching, the automated pairing of coordinates (latitudes; longitudes) and statistical tabulation areas (e.g. census tracts) with street addresses, typically using TIGER/Line or other street data files [1]. The process – which is also known as geocoding – has been described in detail [2,3]. Geocoding usually involves matching addresses to specific street segments then positioning the addresses along the segments assuming an even distribution of street numbers within them. Although this form of geocoding involves linear interpolation and assumptions that can be inappropriate, its inaccuracy may be overlooked in large, population-based studies of associations between spatially interpolated environmental exposures, relevant health outcomes, and their contextual, socioeconomic effect modifiers. Nevertheless, geocoding accuracy is critical when such studies focus on exposure mechanisms that operate over short distances [4].

Although error in assignment of latitudes, longitudes, and census tracts has the potential to bias both estimation of location-specific exposures and socioeconomic contexts within which they occur [5,6], recent studies have reported mean positional errors in commercially geocoded address coordinates between fifty and 300 meters [7-11]. This is a distance over which long-term average ambient air pollution concentrations, meteorological measures and their monitor-to-monitor temporal correlations are relatively constant [12-14]. However, concentrations of traffic-related emissions rapidly fall to ambient levels within comparable distances from street center-lines [15]. Moreover, positional error may be relevant in an even wider range of studies if the previously reported range of distances (50 – 300 m) is an underestimate. Lack of adjustment for potentially important address characteristics suggests that this is a distinct possibility. Population density in the area surrounding an address, for example, is so strongly and inversely associated with positional error that reported distances may be biased by even small differences in the ratio of rural to urban and suburban address matches [16,17]. Positional error also varies markedly with match type, i.e. whether vendors match individual addresses to specific streets or to centers of statistical tabulation areas (centroids) [18], yet to date, most studies have not accounted for these factors.

Published studies of positional error have several additional features that are pertinent in this context. Many restricted their focus to a single geographic setting, address source or geocoding vendor, while those focusing on multiple vendors did not account for among-vendor heteroscedasticity or within-address correlation of positional error [19,20]. Others ignored potential for verification bias [21] and with a notable exception, none examined effects of positional error on exposure measures [7]. Collectively, these observations suggest that the next generation of studies in this area should be designed with generalizability, validity and utility in mind.

To this end, we established three study objectives: (i) to compare multiple geocoding vendors using an identical sample of addresses with known coordinates selected from a broad range of data sources and geographic areas, (ii) to estimate geocoding accuracy and account for address characteristics that affect it using appropriate statistical procedures, and (iii) to estimate effects of observed inaccuracy on individual- and contextual-level exposure measures. We conducted this study to inform research emanating from two studies. The first, *The Environmental Epidemiology of Arrhythmogenesis in WHI *[22], is an ancillary study of electrocardiographic mechanisms linking air pollution and cardiovascular disease in 68,133 U.S. women aged 50–79 years at baseline in the *Women's Health Initiative *(*WHI*) clinical trial [23]. The second, the *Atherosclerosis Risk in Communities *(*ARIC*) study, is a prospective study of cardiovascular disease in 15,792 U.S. men and women aged 45–64 years at baseline [24]. This Institutional Review Board-approved ancillary study complied with all applicable regulations governing human subjects research (University of North Carolina Medical IRB# 03-EPID-12).

## Methods

### Assembling and cleaning addresses

**Figure 1:**
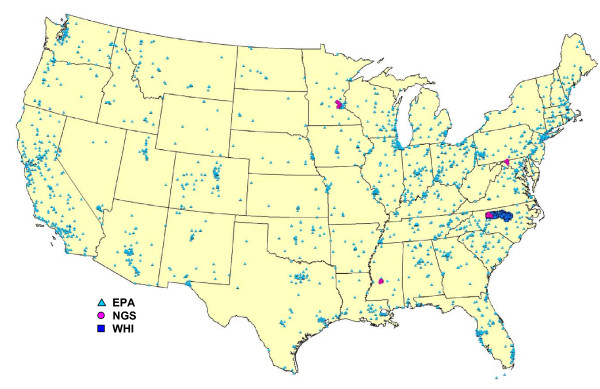
**Location of the 3,615 addresses**. EPA = United States Environmental Protection Agency Air Quality System monitors. NGS = United States National Geodetic Survey stations. WHI = Women's Health Initiative clinical trial participant residential parcels.

We screened seven, publicly available electronic data sources for addresses in areas of the contiguous U.S. containing the 75 WHI and four ARIC exam sites [25-27]. Addresses were eligible for inclusion in this study if they were unique, associated with an established latitude, longitude, street (or route or post office box), city and state; and valid in U.S. Census year 2000. Screening identified 3,615 such addresses: 2,522 of U.S. Environmental Protection Agency (EPA) Air Quality System monitors in the 48 contiguous United States and District of Columbia; 1,050 of WHI clinical trial participants in five counties containing the majority of WHI participants residing in North Carolina (Durham; Forsyth; Guilford; Orange; Wake); and 43 of U.S. National Geodetic Survey (NGS) stations in the four ARIC communities (Forsyth County, NC; Washington County, MD; the city of Jackson, MS; eight suburbs of Minneapolis, MN). We cleaned the addresses (minor edits) when they did not conform to U.S. Postal Service standards [28]. We also used web-based utilities [29-32] to investigate and correct address information (major edits) when it conflicted with that in accompanying field notes (EPA addresses only). If neither condition was met, we did not edit the addresses and flagged them as "unedited". The locations and characteristics of the addresses are described in Figure 1 and Table 1.

**Table 1:** Characteristics of the 3,615 addresses

**Characteristic**	**Stratum or Units**	**n (%) or mean (standard deviation)**
Address Source	EPA	2,522 (70)
	WHI	1,050 (29)
	NGS	43 (1)
Address Type^a^	Complete	2,808 (78)
	No Street Number	460 (13)
	Intersection	347 (10)
Zip Code	Absent	2,359 (65)
	Present	1,256 (35)
Edit	Unedited	1,533 (42)
	Minor	1,392 (39)
	Major	690 (19)
Density^b^	persons/km^2^	1,066 (2,645)
Original Datum^c^	NAD83 or WGS84	1,615 (45)
	Unknown	1,274 (35)
	NAD27	726 (20)

^a^Complete = street number, name, city and state present; No Street Number = street name, city and state present; Intersection = crossing street names, city and state present. ^b^33^rd ^and 67^th ^percentiles = 221 and 920 persons/km^2^. ^c^Of associated coordinates: NAD83 and NAD27 = North American Datum of 1983 and 1927; WGS84 = World Geodetic System of 1984.

### Spatial data quality

Coordinates in decimal degrees with at least six significant digits after the decimal point accompanied all addresses. EPA coordinates were established according to a federal accuracy standard of < 25 m [33], NGS coordinates, according to a federal standard < 10 m [34], and WHI coordinates, by applying a spatial routine that determines center points of residential land parcels on digital maps (adapted from O'Rourke [35]). The median accuracy of the latter method approximates that of high resolution aerial photography, 8 to 15 m depending on population density [16]. These coordinates and their associated block group, tract, and county identifiers (U.S. Census 2000 Federal Information Processing Standards [FIPS] codes) served as the criterion standards against which the accuracy of vendor-assigned geocodes was measured.

### Geocoding addresses and estimating accuracy

We submitted the addresses to four well-known vendors (A-D) frequently contracted by epidemiologists for geocoding and related services or products (Table 2). We label the vendors generically in this paper to mask their identity, a practice consistent with our current data use agreements and previously implemented in similar contexts [5,7,20]. To examine whether editing introduced error, we also submitted unedited versions of the edited EPA addresses to one of the vendors. We estimated the accuracy of geocodes assigned by the vendors using three previously defined measures: (i) the address match rate (%), i.e. percentage of all addresses to which a given vendor assigned a latitude, longitude and FIPS code; (ii) the concordance (%) between vendor-assigned and criterion standard FIPS codes; and (iii) the distance in meters between vendor-assigned and criterion standard coordinates, as measured using the Haversine spherical Earth formula (***ρ***) [20]. We based the measures on analyses of spatial data that we transformed, when necessary, to a standard geographic coordinate system using ArcGIS^® ^9.0.

**Table 2:** Characteristics of the four vendors

**Vendor**	**CASS**	**Street Offset**	**Corner Inset**	**Street Data Files**			**Scheduled Data File Updates**	**Original Datum^**a**^**	**Manual Address Cleaning**^**b**^
				**TIGER**	**USPS**	**Other**			
A	Yes	40 ft	Yes	2002	2004	Yes	4×/yr	WGS84	No
B	No	5 ft	Yes	2002	2004	Yes	4×/yr	NAD83	No
C	Yes	50 ft	No	2002	2004	Yes	6×/yr	NAD83	Yes
D	No	0 ft	No	2002	2003	No	2×/yr	NAD83	No

^a^Of assigned coordinates: NAD83 = North American Datum of 1983. WGS84 = World Geodetic System of 1984. ^b^After initial processing by geocoding software. CASS = Address standardization certified by the United States Postal Service National Customer Support Center Certification Program, Coding Accuracy Support System. TIGER = Topologically Integrated Geographic Encoding and Referencing (TIGER/Line^®^) file. USPS = United States Postal Service files e.g. the city-state, ZIP+4^® ^and ZIPMove products.

### Analysis of variance

We used analysis of variance (ANOVA) to quantify the variation in ***ρ ***(log-transformed to satisfy the assumption of Gaussian errors) among vendors, before and after controlling for characteristics that affect geocoding accuracy: address source (EPA; WHI; NGS), address type (complete; no street number; intersection), zip code (present; absent), editing (unedited; minor; major), population density of the associated census tract (persons/km^2^), and original coordinate datum (North American Datum of 1983 [NAD83] or World Geodetic System of 1984 [WGS84]; North American Datum of 1927 [NAD27]; unknown). In this context, "no street number" includes rural route and post office box addresses. After testing for effect modification (significance of the interaction between vendor and match type), we stratified ANOVA models. We computed adjusted, least-square means among vendors using weights that were proportional to the observed distribution of covariates in our dataset. We back-transformed predicted values to the original scale as follows: , where  and  were the vendor-specific least square means and variances of log ***ρ***, the latter estimated from the residuals. We used logistic regression to estimate the odds ratios and 95% confidence intervals (OR, 95% CI) for address match and census tract concordance among vendors, before and after adjustment for the same address characteristics used in the ANOVA models. We arbitrarily chose vendor C as a basis for comparison in these logistic models.

### Within-address dependence and among-vendor heteroscedasticity of *ρ*

Recognizing that the above analyses failed to account for the observed dependence of coordinates assigned to the same address by different vendors and the heterogeneity of variances across vendors (among centroid-type matches), we repeated analyses using mixed effects models. This modeling framework allowed simultaneous specification of the within-address dependence and among-vendor heteroscedasticity of ***ρ***. Assuming values of ***ρ ***provided by different vendors were equally correlated, we used a compound symmetric (exchangeable) covariance structure. We were not interested in testing hypotheses concerning the variances and covariances of the within-address covariance matrix. We simply considered them as nuisance parameters needing to be controlled. We also considered the addresses as a random sample of a larger defined population, and the sample of vendors as fixed. Inferences therefore pertain to the four vendors.

### Application

We examined the effects of geocoding error over the observed range of ***ρ ***in a 5% random sample of street-type address matches (n = 2,608) and a census of centroid-type address matches (n = 2,671) from *The Environmental Epidemiology of Arrhythmogenesis in WHI*, 1999–2002 [36]. Briefly, we displaced the coordinates associated with each address at random over a uniform distribution of ***θ ***(range, 0–360°) and lognormal distributions of ***ρ ***with means and standard deviations approximating the range of values observed in this context. We used ArcGIS^® ^9.0 to assign the original and displaced coordinates to year 2000 U.S. Census tracts and to estimate the distance between the coordinates and the nearest interstate, U.S., or state highway or major traffic thoroughfare at that time. Consistent with prior literature, we dichotomized this distance at 100 meters to create a simple proxy for traffic-related air pollution exposure [15,37]. Then we examined the effect of displacement on this proxy, exposure misclassification rates and census tract concordance. We completed all analyses using the SAS, Version 9.1 software package.

## Results

**Figure 2:**
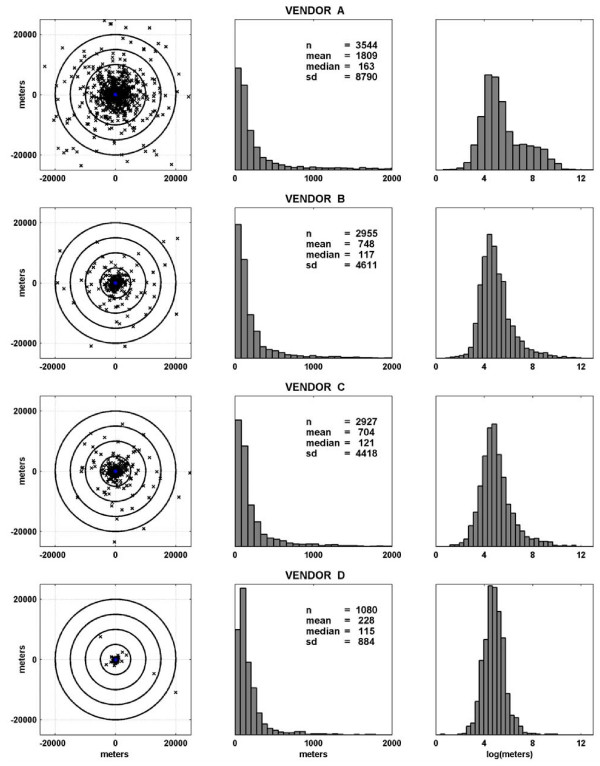
**Distribution of the spherical distance in meters (*ρ*) between criterion standard and vendor-assigned coordinates, by vendor**. Column I: Scatterplots in which **X**s and center points represent vendor-assigned and criterion standard coordinates, respectively. Columns II and III: Normalized frequency histograms before (II) and after (III) log-transformation. Columns I and II exclude outlying values to allow equal cross-vendor scaling of axes in meters. n = sample size. sd = standard deviation.

Door-to-door return times and geocoding costs were generally reasonable across vendors: range, 2–5 business days and $16–$25 per 1,000 addresses. However, analyses of the edited address database revealed large differences among vendors A-D in address match rate (98%; 82%; 81%; 30%), census tract concordance (85%; 88%; 87%; 98%) and mean ***ρ ***(1809; 748; 704; 228 m) (Table 3 and Figure 2). Address match rate and census tract concordance were relatively high and mean ***ρ***, relatively low among WHI, complete, zip-coded, unedited, and urban or suburban addresses; addresses with NAD83 or WGS84 criterion standard coordinates; and street-type matches (Table 4).

**Table 3:** Accuracy of geocodes assigned by the four vendors

**Vendor**	**Match Rate**			**Concordance**			***ρ***^**c**^
	**Overall**^**a**^	**Street**	**Centroid**^**b**^	**Block Group**	**Tract**	**County**	
A	98%	79%	20%	77%	85%	99%	1809 (8790)
B	82%	78%	4%	83%	88%	99%	748 (4611)
C	81%	77%	4%	81%	87%	99%	704 (4418)
D	30%	30%	0%	97%	98%	100%	228 (884)

^a^Due to rounding, may differ from the sum of street- and centroid-type match rates.

^b^Geographic or delivery-weighted center of a statistical tabulation area, e.g. U.S. Census tract. ^c^Spherical distance in meters between criterion standard and vendor-assigned coordinates (mean [standard deviation]).

**Table 4:** Overall match rate, census tract concordance and *ρ*^**a**^, by address and match characteristics

**Characteristic**	**Stratum**	**Match Rate**	**Census Tract Concordance**	***ρ***^**a**^
Address Source	EPA	62%	47%	1,619 (7,904)
	NGS	88%	72%	1,125 (3,711)
	WHI	98%	97%	159 (409)
Address Type	No Street Number	28%	8%	5,111 (6,150)
	Intersection	60%	43%	1,259 (6,270)
	Complete	82%	73%	793 (6,063)
Zip Code	Absent	60%	45%	1,609 (8,205)
	Present	96%	92%	376 (1,634)
Edit	Major	59%	45%	2,622 (10,029)
	Minor	70%	58%	828 (3,833)
	Unedited	81%	73%	688 (5,877)
Density^b ^(persons/km^2^)	Rural, 0–221	65%	54%	2,069 (8,280)
	Suburban, 222–920	79%	71%	566 (6,172)
	Urban, ≥ 920	74%	60%	485 (2,319)
Datum^c^	Unknown	60%	43%	1,600 (8,612)
	NAD27	64%	51%	1,475 (6,619)
	NAD83 or WGS84	87%	81%	590 (3,961)
Match Type	Centroid	100%	34%	5,331 (9,207)
	Street	100%	90%	607 (5,577)

^a^Spherical distance in meters between criterion standard and vendor-assigned coordinates (mean [standard deviation]). ^b^Stratified at the 33^rd ^and 67^th ^percentiles. ^c^Original datum of coordinates. NAD27 and NAD83 = North American Datum of 1927 and 1983. WGS84 = World Geodetic System of 1984.

In analyses restricted to vendors with minimally acceptable match rates (A-C), among-vendor differences in mean ***ρ ***were small for street-type matches (293; 287; 288 m). In contrast, differences between centroid-type matches were substantial in some vendor contrasts, but not others (6375; 4854; 5524 m), p for interaction < 10^-4^. Adjustment for address characteristics, within-address correlation and heteroscedasticity of ***ρ ***reduced the mean and standard deviation of ***ρ ***(Table 5). The pattern of adjusted mean ***ρ ***among vendors reflected that of the adjusted odds of an address match: it was higher for vendor A versus C (OR = 66, 95% CI: 47, 93), but not B versus C (OR = 1.1, 95% CI: 0.9, 1.3). The adjusted odds of census tract concordance were, by comparison, no higher for vendor A versus C (OR = 1.0, 95% CI: 0.9, 1.2) or B versus C (OR = 1.1, 95% CI: 0.9, 1.3) (Table 6).

**Table 5:** Spherical distance in meters (*ρ*) between criterion standard and vendor-assigned coordinates (mean [standard deviation]), by match type and vendor

**Match Type**	**Vendor**	***ρ***			
		**Unadjusted**	**Adjusted**^**a**^	**Within**^**a**,**b**^	**Hetero**^**a-c**^
Street	A	293 (564)	272 (476)	280 (492)	NA
	B	287 (545)	262 (438)	268 (447)	NA
	C	288 (551)	266 (456)	275 (471)	NA
Centroid	A	6,375 (10,437)	6,194 (9,473)	5,630 (8,576)	5,497 (8,345)
	B	4,854 (27,279)	3,663 (15,948)	4,230 (18,730)	4,303 (19,185)
	C	5,524 (34,703)	3,298 (13,068)	3,900 (15,943)	4,210 (17,638)

^a^For address source, type, zip code, edit, population density (persons/km^2^) and datum.

^b^Also adjusted for within-address correlation of ***ρ***. ^c^Additionally adjusted for among-vendor heteroscedasticity of ***ρ ***(see methods). NA = not applicable.

**Table 6:** Odds ratios (95% confidence intervals) for overall address match and census tract concordance, by vendor

	**Overall Address Match**		**Census Tract Concordance**	
**Vendor**	**Unadjusted**	**Adjusted**^**a**^	**Unadjusted**	**Adjusted**^**b**^
A	12 (9, 15)	66 (47, 93)	0.8 (0.7, 0.9)	1.0 (0.9, 1.2)
B	1.1 (0.9, 1.2)	1.1 (0.9, 1.3)	1.1 (0.9, 1.2)	1.1 (0.9, 1.3)
C	1.0	1.0	1.0	1.0

^a^Adjusted for address source, type, zip code, edit, population density, and datum. ^b^Also adjusted for match type.

Further restricting analyses to records successfully geocoded by all vendors A-C attenuated mean ***ρ ***and its pattern of differences among them. Match rate and census tract concordance were much lower, and mean ***ρ***, much higher in analyses of the unedited versus edited EPA addresses (data not shown).

The percent of street-type address matches < 100 meters away from the nearest highway was relatively constant across mean ***ρ ***(Table 7). This apparent absence of misclassification was related to counter-balancing effects of approximately equal false positive and false negative rates at values of mean ***ρ ***between 150 and 600 meters. Together, they accounted for a 14% increase in the total error rate over the same range. This increase was accompanied by a 20% decrease in census tract concordance.

**Table 7:** Effect of mean *ρ*^a ^on classification of distance to the nearest highway^b^, exposure misclassification rates^c ^and census tract concordance^d^

**Match**	**Mean**	**Distance**	**Misclassification Rates**			**Census Tract**
**Type**	***ρ***	**< 100 m**	**False +**	**False –**	**Total**	**Concordance**
Street	0	27%	0%	0%	0%	100%
	150	29%	8%	6%	15%	90%
	300	26%	11%	11%	22%	82%
	600	27%	15%	14%	29%	70%
Centroid	0	32%	0%	0%	0%	100%
	2,500	19%	9%	22%	31%	66%
	5,000	16%	9%	25%	33%	55%
	10,000	14%	8%	26%	34%	42%

^a^Spherical distance in meters between criterion standard and vendor-assigned coordinates. Standard deviation of ***ρ ***= 500 and 15,000 meters for street- and centroid-type matches, respectively. ^b^Interstate, U.S., or state highway or major traffic thoroughfare. ^c^False + indicates misclassification of the unexposed (≥ 100 m) as exposed (< 100 m). False – indicates misclassification of the exposed as unexposed. The sum of false + and – error rates may not equal the total error rate due to rounding. ^d^Percent of census tracts matching those in the datasets without positional error (***ρ ***= 0). Based on a 5% random sample of street-type address matches (n = 2,608) and a census of centroid-type address matches (n = 2,671) in The Environmental Epidemiology of Arrhythmogenesis in WHI, 1999–2002.

In contrast, the percent of centroid-type address matches classified as < 100 meters away from the nearest highway was approximately two-fold higher at zero versus non-zero values of mean ***ρ ***(Table 7). This finding was related to the two- to three-fold excess of false negative versus false positive rates at values of mean ***ρ ***between 2,500 and 10,000 meters. The total error rate increased by 3% and census tract concordance decreased by 24% over the same range.

## Discussion

Persistent concerns about the potential effects of inaccurate geocoding on spatially interpolated environmental exposures, exposure-outcome associations, and their contextual effect modifiers have stimulated interest in the positional error of commercially geocoded address coordinates. However, studies of the topic have often reported average positional errors in the range of fifty to 300 meters [6-9,16-20]. Although these reports have reduced such concerns, few studies have focused on multiple geographic areas, address sources and vendors; adjusted accuracy measures for important address and methodological characteristics; and estimated the influence of inaccuracy on individual- and contextual-level exposure measures. The generalizability, validity and utility of these estimates is therefore unclear.

We addressed this issue in a Women's Health Initiative ancillary study, *the Environmental Epidemiology of Arrhythmogenesis in WHI*, by submitting addresses selected from a broad range of data sources and geographic areas to four well-known vendors often contracted by epidemiologists for geocoding and related services or products (at the time of submission, they had been in business for a combined total of > 35 years, employed > 650 persons, and reported > $50 million of annual sales [38]). We then examined differences between vendors in address match rate, census tract concordance and mean ***ρ***.

We found that geocoding error depends on measures used to evaluate it and vendor. More specifically, vendors matching lower proportions of addresses geocoded them with higher spatial accuracy, i.e. higher census tract concordance and lower mean ***ρ***. We also found that that geocoding error depends on address characteristics. Mean ***ρ***, for example, was relatively high among EPA, incomplete, unzip-coded, edited and rural addresses; addresses with NAD27 criterion standard coordinates; and in particular, centroid-type address matches. After stratifying by match type, then adjusting for the remaining address characteristics and other methodological factors, mean ***ρ ***remained twenty times higher among vendor A's centroid- versus street-type address matches. The adjusted odds of an address match also remained more than sixty times higher for vendor A than either B or C. Lastly, by randomly displacing address coordinates over the range of mean ***ρ ***observed in this context, we found that traffic-related pollution exposure misclassification rates increased and census tract concordance decreased with corresponding increases in mean ***ρ***.

Considered together, these findings suggest that vendor selection presents a trade-off between potential for missing data and error in estimating spatially defined attributes such as environmental exposure and socioeconomic context. They also indicate that the trade-off can be quite unbalanced. Vendor D, for example, matched an unacceptably low proportion of addresses, but geocoded them with a singularly high level of spatial accuracy. Moreover, the observed association between missing data and positional error across vendors suggests that while vendors may be targeting different points along the trade-off spectrum, they tend to retain observations that are likely to have positional errors. Deleting these observations would of course translate into reduced potential for bias due to individual- and contextual-level exposure measurement error, but it remains unclear whether vendors can increase data accuracy without compromising its availability.

Although these findings may have greater generalizability, validity and utility than those previously reported, our criterion standards may have been imperfect. Interpretation must therefore recognize potential for bias due to the elusiveness of a definitive criterion standard. Indeed, match rate and concordance may have been overestimated and mean ***ρ***, underestimated because using imperfect criterion standards tends to artificially inflate accuracy [21].

Since errors in accuracy measures vary with errors in imperfect criterion standards, we therefore edited addresses when they failed to conform to U.S. postal standards or conflicted with field notes. Editing was intended to reduce misspelled, misspaced or inappropriately abbreviated state, street suffix or secondary unit designators like "apartment" [28]. Though well-intentioned, editing may have introduced error instead of reducing it. Mindful of this possibility, we submitted both the unedited and edited versions of EPA addresses for geocoding. We found that, on average, match rate and census tract concordance were much higher and mean ***ρ***, much lower in analyses of the edited versus unedited versions of the database. This finding confirmed that, on average, editing tended to correct addresses and thereby reduce error in accuracy measures, but as a precaution, we also adjusted measures of accuracy for edit type.

Even after editing addresses, our criterion standards may have contained erroneous coordinates of EPA monitors, NGS stations and WHI participants. Such errors have been identified, for example, within EPA databases of environmental hazards in South Carolina [39]. Although theses errors vary across data sources, among states and over time, their potential existence in this context is no less a concern. The EPA implemented its Locational Data Policy in 1991 in response to concerns of this sort. It stipulated adoption of uniform methods, use of global positioning systems and collection of monitor coordinates according to a Federal Interagency Coordinating Committee on Digital Cartography accuracy standard of 25 meters [33]. Five years later, the EPA also launched its Locational Data Improvement Project as a vehicle for further improvement in the accuracy of its databases [40]. Moreover, the NGS adheres to a stricter, 1998 Federal Geographic Data Committee standard of less than ten meters [34] – a distance identical to that between parcel center points and true residential locations in urban settings and somewhat less than that in rural areas [16]. We also adjusted measures of accuracy for differences among address sources despite these reassurances.

Interpretation of the findings reported here must also consider the challenges inherent in disentangling the general effect of vendor and the specific effect of a given geocoding method. Street offset – the perpendicular distance between vendor-assigned coordinates and the corresponding street centerline – serves as an illustrative example. Although researchers are often troubled by vendors' underlying assumption that this distance is equal for all addresses, a different study design would have been required to discriminate effects of vendor and offset because as a default, vendors A-D used distinct offsets between zero and fifty feet. However, a repeated-measures design – one in which the same addresses would have been geocoded repeatedly by the same vendors using different offsets – was not feasible: the option of changing defaults was not uniformly available among vendors A-D. Even if it had been, prior reports suggesting that the contribution of offset to geocoding accuracy is rather modest within the narrow range of defaults observed in this context are reassuring [11,16].

## Conclusion

With these caveats in mind, we conclude that informed selection of geocoding practices and approaches to data analysis involves estimating potential for, balancing the trade-off between, and when appropriate, adjusting for the effects of missing data and error in spatially defined attributes. We suggest beginning this process by submitting (masked) addresses associated with high quality criterion standard coordinates in a given study area to geocoding vendors, estimating the accuracy of vendor-assigned coordinates, and selecting vendors that balance the tradeoff between missing data and error in ways that best meet study needs. If edited and unedited forms of the same address are included in the geocoded data set, address cleaning procedures – which should (but may not) be standardized – can be simultaneously evaluated.

Comparing the limitations of methods commonly used to analyze incomplete data with those used to adjust for positional or exposure measurement error may help prioritize individual study needs in advance [41-44]. Basic algebra, for instance, can be used to adjust associations for exposure measurement error [44]. Consider the cell counts observed in a hypothetical case-control study of the association between distance to the nearest highway and coronary heart disease mortality (Table 8). The sensitivity (se) and specificity (sp) of the 100 m distance classification at mean ***ρ ***= 150 m can be calculated from the corresponding false negative (fn) and false positive (fp) rates in Table 7:

**Table 8:** Cell counts from a hypothetical case-control study of the association between distance to the nearest highway and coronary heart disease mortality

**Distance**	**Case**	**Non-Case**
< 100 m	a* = 88	b* = 108
≥ 100 m	c* = 137	d* = 294

OR* = (a* × d*) ÷ (b* × c*) = 1.8

se = 1 - fn = 1 - 0.06 = 0.94

sp = 1 - fp = 1 - 0.08 = 0.92

Under non-differential misclassification, the corrected cell counts are

a = (a* - 0.08 × (a* + c*)) ÷ (0.94 + 0.92 - 1) = 81.40

b = (b* - 0.08 × (b* + d*)) ÷ (0.94 + 0.92 - 1) = 88.19

c = (a* + c*) - a = 143.61

d = (b* + d*) - b = 313.81

and in the absence of confounding, the corrected odds ratio is

OR = (a × d) ÷ (b × c) = (81.40 × 313.81) ÷ (88.19 × 143.61) = 2.0

This odds ratio is more extreme than its uncorrected counterpart, OR* (Table 8), which is biased toward the null. Its corrected probability distribution can be estimated using Monte Carlo simulation [45].

However, the magnitude of exposure measurement error in a continuous variable such as distance to the nearest highway may not vary directly with the magnitude of a given exposure-outcome association. When it is independent of disease status, the resulting misclassification of commonly used exposure categories (e.g. distance < or ≥ 100 meters) may be differential and vary in unanticipated ways. Seemingly appropriate adjustments may also be inaccurate even when this type of misclassification is non-differential [43]. Such adjustments must therefore be applied with caution.

Nonetheless, uninformed selection of geocoding practices and data analysis appears to be a less desirable alternative, particularly in studies of exposure mechanisms operating within short distances. The positional errors reported here suggest that "short" should be defined as less than 280 meters for potentially geocodable addresses matched at the street level and less than 5.5 kilometers for those matched at the centroid level by well-known vendors with minimally acceptable match rates. Critical distances, though, may be substantially lower given the non-negligible misclassification rates we observed when mean ***ρ ***was approximately one-half as large as these values. More accurate geocoding methods that involve global positioning or parcel matching can be used to reduce potential for bias in studies requiring such high levels of spatial resolution [2,16]. Use of the latter method is expected to grow over time as high quality, parcel-level databases become more uniformly available across larger study areas.

## Abbreviations

ARIC Atherosclerosis Risk in Communities

CASS Coding Accuracy Support System

EPA Environmental Protection Agency

FIPS Federal Information Processing Standards

NAD27 and NAD83 North American Datum of 1927 and 1983

NGS National Geodetic Survey

TIGER Topologically Integrated Geographic Encoding and Referencing

USPS United States Postal System

WHI Women's Health Initiative

WGS84 World Geodetic System of 1984

## Competing interests

The author(s) declare that they have no competing interests.

## Authors' contributions

EAW conceived of the study, designed it, and drafted the manuscript. PMQ assembled and analyzed the data, and helped draft the manuscript. RLS directed the statistical analysis and helped draft the manuscript. DJC helped direct the statistical analysis and draft the manuscript. DL helped design the study and draft the manuscript. ACH directed handling of geographic data and helped draft the manuscript. GH helped design the study and draft the manuscript.
